# The m6A writers regulated by the IL-6/STAT3 inflammatory pathway facilitate cancer cell stemness in cholangiocarcinoma

**DOI:** 10.20892/j.issn.2095-3941.2020.0661

**Published:** 2021-08-04

**Authors:** Hua Ye, Tianqi Chen, Zhancheng Zeng, Bo He, Qianqian Yang, Qi Pan, Yueqin Chen, Wentao Wang

**Affiliations:** 1 Department of Hepatobiliary, Sun Yat-sen Memorial Hospital, Sun Yat-sen University, Guangzhou 510120, China; 2 Key Laboratory of Gene Engineering of the Ministry of Education, School of Life Sciences, Sun Yat-sen University, Guangzhou 510275, China; 3 Department of Anesthesiology, Sun Yat-sen Memorial Hospital, Sun Yat-sen University, Guangzhou 510120, China

**Keywords:** Cholangiocarcinoma, IL-6, cell stemness, N6-methyladenosine (m6A), IGF2BP2

## Abstract

**Objective:**

Investigation of the regulatory mechanisms of cell stemness in cholangiocarcinoma (CCA) is essential for developing effective therapies to improve patient outcomes. The purpose of this study was to investigate the function and regulatory mechanism of m6A modifications in CCA cell stemness.

**Methods:**

Interleukin 6 (IL-6) treatment was used to induce an inflammatory response, and loss-of-function studies were conducted using mammosphere culture assays. Chromatin immunoprecipitation, polysome profiling, and methylated RNA immunoprecipitation analyses were used to identify signaling pathways. The *in vitro* findings were verified in a mice model.

**Results:**

We first identified that m6A writers were highly expressed in CCAs and further showed that STAT3 directly bound to the gene loci of m6A writers, showing that IL-6/STAT3 signaling regulated expressions of m6A writers. Downregulating m6A writers prevented cell proliferation and migration *in vitro* and suppressed CCA tumorigenesis *in vivo*. Notably, the knockdown of m6A writers inhibited CCA cell stemness that was triggered by IL-6 treatment. Mechanistically, IGF2BP2 was bound to CTNNB1 transcripts, significantly enhancing their stability and translation, and conferring stem-like properties. Finally, we confirmed that the combination of m6A writers, IGF2BP2, and CTNNB1 distinguished CCA tissues from normal tissues.

**Conclusions:**

Overall, this study showed that the IL-6-triggered inflammatory response facilitated the expressions of m6A writers and cell stemness in an m6A-IGF2BP2-dependent manner. Furthermore, the study showed that m6A modification was a targetable mediator of the response to inflammation factor exposure, was a potential diagnostic biomarker for CCA, and was critical to the progression of CCA.

## Introduction

Cholangiocarcinoma (CCA) is a common biliary malignant tumor, whose incidence is increasing annually, especially in China^1–4^. CCA is difficult to diagnose and cure, partly due to unknown molecular mechanisms and the underlying mechanism of cancer cell self-renewal^1,2^. Studies have suggested that chronic inflammation plays important roles in CCA pathogenesis, and that a large number of inflammatory cytokines are expressed because of chronic inflammatory reactions^1,5–7^. We have previously reported that microRNA clusters, let-7c/miR-99a/miR-125b, regulated CCA progression and stem-like properties through the IL-6/STAT3 pathway, leading to the promotion of CCA cell transformation, which suggested that the inflammatory response facilitated cancer cell stemness and tumor progression^8^. However, how these inflammatory factors regulate cancer cell stemness and tumor progression in CCA remains largely unknown. A more comprehensive understanding of the mechanisms by which inflammatory factors drive cancer progression and metastasis is therefore needed to develop effective therapies^9,10^.

N6-methyladenosine (m6A) is the most abundant RNA modification in eukaryotic cells^11,12^. Accumulated evidence has indicated that m6A plays critical roles in multiple biological processes, and aberrant m6A modification is closely associated with cancers^12–16^. The breakthrough in the discovery and understanding of m6A writers, erasers, and readers, together with the development of high-throughput assays, have helped to elucidate the biological functions and the underlying mechanisms of m6A^12,17,18^. For example, methyltransferase 3 (METTL3) serves as an oncogene to promote tumorigenesis by enhancing m6A modification and the translation of BCL2 and PTEN in acute myeloid leukemia^17^, and methyltransferase 14 (METTL14) acts as a suppressor to inhibit tumor metastasis by promoting miR-126 processing in an m6A-dependent manner in hepatocellular carcinoma (HCC)^18^. Recent studies have also indicated that m6A modification plays critical roles in cell self-renewal and tumorigenesis^17,19–21^. However, whether m6A modification regulates the inflammatory response in CCA cell stemness remains unknown.

In this study, we aimed to identify the role and regulatory mechanism of the m6A modification in the CCA cell stemness and inflammatory response. We first showed that m6A writers were highly expressed in CCA tumor tissues and had distinct expression patterns in intra-hepatic CCA (ICC) and extra-hepatic CCA (ECC) samples, suggesting that they could potentially identify the location of cancerous lesions in CCA cells and discriminate between different CCA subtypes. We showed that the expressions of m6A writers in CCA cells were upregulated by cytokine IL-6 upon STAT3 directly binding to the m6A writer gene region, making m6A a potential targetable mediator in response to inflammation factor exposure. Specifically, m6A modification significantly enhanced the stability and translation of stem genes, including CTNNB1, and facilitated the stem-like properties of CCA cells, highlighting its crucial roles in the self-renewal of CCA cells. This study therefore provided insight into the inflammatory response and suggested that CCA stemness gene expression was regulated through an m6A-dependent pathway.

## Materials and methods

### Patients

Human CCA and peritumoral (designated as normal) tissues were obtained with informed consent between 2017 and 2019 from Sun Yat-sen Memorial Hospital. Sample collection was approved by the Hospital’s Protection of Human Subjects Committee (Approval No. 2017126). Thirty-eight pairs of normal peritumoral specimens and pathologically diagnosed biopsy specimens were obtained from the same CCA patients who underwent surgical resections. The clinicopathological characteristics of the CCA patients are summarized in **Supplementary Table S1**.

### Cell lines and cell culture

SK-Cha-1, MZ-Cha-1, and RBE human CCA cells were kindly provided by Dr. Chundong Yu (Xiamen University, Fujian, China), and human embryonic kidney 293 cells were purchased from the American Type Tissue Collection (Manassas, VA, USA) and were cultured in RPMI-1640 (HyClone, Logan, UT, USA) and DMEM (HyClone), respectively, with 10% fetal bovine serum (Gibco, Gaithersburg, MD, USA). All cells were cultured at 37 °C in a 5% CO_2_ atmosphere.

### RNA isolation and quantitative real-time PCR (RT-PCR)

Total RNA was extracted using TRIzol reagent (Invitrogen, Carlsbad, CA, USA) in accordance with the manufacturer’s instructions. RNA was reverse transcribed into cDNA using the RT reagent Kit RR047A (Takara, Shiga, Japan) and followed by real-time PCR with a SYBR Premix ExTaq Real-time PCR Kit (Takara). All gene expression levels were normalized to glyceraldehyde 3-phosphate dehydrogenase (GAPDH). Oligonucleotide sequences are listed in **Supplementary Table S2**.

### Cell transfection and shRNA transduction of cells

Lipofectamine 2000 (Invitrogen) was used to perform the transient transfections of recombinant vectors and siRNAs. The pGreenPuro™ shRNA was packaged into lentiviruses using the Lentivector Expression Systems (System Biosciences, Heidelberg, Germany) consisting of pPACKH1-GAG, pPACKH1-REV, and pVSV-G^22^.

For stable expression assays, 2 × 10^5^ SK-Cha-1 and RBE cells were prepared for each transfection system. The cells were centrifuged and resuspended in 300 µL virus suspension, followed by incubation at 37 °C and 5% CO_2_ for 24 h. Then, the cells were centrifuged, washed, and resuspended in fresh medium containing 1.5 µg/mL puromycin and 1% penicillin-streptomycin. To confirm target knockdown, the cells were collected for qRT-PCR analysis. The siRNA and shRNA sequences are listed in **Supplementary Table S2**.

### CCK-8 cell proliferation assay

Cells first transfected with m6A writer siRNAs were seeded at a density of 20,000 cells per well in 100 µL of complete medium in 96-well plates. Then, 10 µL of the Cell Counting Kit-8 (Dojindo Molecular Technologies, Nanjing, China) reagent was added, and the cells were cultured for another 2 h. Absorbance was measured using a VICTOR™ X5 Multilabel Plate Reader (Perkin Elmer, San Jose, CA, USA) at wavelengths of 480 and 630 nm at 0, 24, 48, 72 and 96 h.

### Scratch wound healing assay

The siRNA-transfected SK-Cha-1 cells were cultured in 24-well plates for 24 h. Linear wounded tracks were generated with sterile, 10 µL pipettes. The scratched cells were then rinsed twice with phosphate-buffered saline (PBS) to remove non-adherent cells, and fresh culture medium was added. Photographs of the centers of gaps were taken using a phase-contrast microscope and the same magnification of 100×. The cell migrations at 0 and 24 h after scratching were evaluated by determining the wound distance at random wound gap locations. The closure area of the wound was calculated as follows: migration area (%) = (M_0_ – M_24_)/M_0_ × 100, where M_0_ represents the initial wound area, and M_24_ represents the remaining area of wound at the 24 h time point^23,24^.

### Protein extraction and immunoblotting

Total protein was extracted from cell samples using RIPA lysis buffer (Beyotime Biotechnology, Beijing, China) supplemented with 1× complete ULTRA protease inhibitor (Roche, Basel, Switzerland) according to the manufacturer’s instructions. Proteins were resolved by 7.5%, 10%, or 12% bis-tris polyacrylamide gels and were transferred to polyvinylidene fluoride membranes and then blocked and probed with the appropriate antibodies overnight at 4 °C. Finally, the membranes were incubated with horseradish peroxidase-conjugated secondary antibodies at room temperature for 1 h and visualized with an enhanced chemiluminescence detection system. Detailed information of the antibodies is found in **Supplementary Table S3**.

### Chromatin immunoprecipitation (ChIP)

ChIP analyses were performed on chromatin extracts from SK-Cha-1 and MZ-Cha-1 cells using a Magna ChIP™ G-Chromatin Immunoprecipitation Kit (Merck Millipore, Darmstadt, Germany) with STAT3 antibody according to the manufacturer’s standard protocol. In this assay, samples incubated with rabbit IgG served as the negative control. The fold enrichment of STAT3 was quantified by quantitative RT-PCR and calculated relative to input chromatin. The primers used for ChIP-qPCR analysis are listed in **Supplementary Table S2**.

### The m6A dot blot

Total cellular RNA was first denatured in a 3-fold volume of RNA incubation buffer (65.7% formamide, 7.77% formaldehyde, and 1.33× MOPS) at 65 °C for 5 min, and then mixed with a 1-fold volume of 20× SSC. After UV crosslinking, the membrane was stained with 0.02% methylene blue in 0.3 M sodium acetate. The membrane was then washed with 1× PBST buffer, blocked with 5% nonfat milk in PBST, and incubated with anti-m6A antibody overnight at 4 °C. After incubating with horseradish peroxidase-conjugated anti-rabbit IgG secondary antibody (Santa Cruz Biotechnology, Santa Cruz, CA, USA), the membrane was visualized by ECL (Thermo Fisher Scientific, Waltham, MA, USA) in a dark room.

### RNA immunoprecipitation (RIP)

For RIP assays, the proteins were first lysed with cell lysis buffer supplemented with Thermo Scientific™ Halt™ Protease Inhibitor Cocktail (Thermo Fisher Scientific). Then, IGF2BP2 antibody was used along with an EZ-Magna RIP™ RNA-Binding Protein Immunoprecipitation Kit (Merck Millipore) according to the manufacturer’s instructions. Finally, the fold enrichment of IGF2BP2 was quantified by quantitative RT-PCR and calculated relative to the input.

### The methylated RNA immunoprecipitation (MeRIP) assay

Total RNA was first isolated before mRNA enrichment using the GenElute mRNA miniprep kit (Sigma-Aldrich, St. Louis, MO, USA). The mRNA of the sample was further treated with m6A MeRIP according to the protocol of the Magna MeRIP™ m6A Kit Transcriptome-wide Profiling of N6Methyladenosine (EMD Millipore, Hayward, CA, USA). Finally, m6A enrichment was quantified by quantitative RT-PCR and calculated relative to the input.

### The RNA stability assay

SK-Cha-1 cells were treated with actinomycin D at a final concentration of 5 mg/mL for 5 and 10 h prior to cell collection. Total RNA was extracted by miRNeasy Kit (Qiagen, Hilden, Germany) and analyzed by RT-PCR.

### Polysome profiling

The sh-NC and sh-IGF2BP2 SK-Cha-1 cells (3.5 × 10^7^ cells) were treated with 200 µg/mL cycloheximide (CHX) for 15 min, and lysed in lysis buffer (20 mM Tris, pH 7.4, 15 mM MgCl_2_, 200 mM KCl, and 1% Triton X-100) supplemented with 40 U/mL RNasin (Promega, Madison, WI, USA), 100 µg/mL CHX, and 1 mM dithiothreitol. Cell lysates were centrifuged at 13,000 rpm at 4 °C for 10 min and then the supernatant ultracentrifuged using a SW41 rotor (Beckman, Brea, CA, USA) at 36,000 rpm at 4 °C for 3 h, then loaded onto 10%–45% sucrose gradients. The absorbance at 260 nm was determined using a BioComp Piston Gradient Fractionator (BioComp Instruments, Fredericton, Canada) equipped with a Bio-Rad Econo UV Monitor (Bio-Rad, Hercules, CA, USA). The corresponding isolated fractions were further used for RNA in TRIzol reagent (Invitrogen) and then for RT-qPCR. The relative distribution of mRNA in each fraction was normalized by the total abundance of mRNA in all fractions marked as 100%^25^.

### Mammosphere cultures

Cells (1,500 cells/mL) were cultured for 8 days in serum-free DMEM-F12 (BioWhittaker, Radnor, PA, USA) supplemented with B27 (1:50; Invitrogen), 20 ng/mL EGF (Invitrogen), 20 ng/mL bFGF (Invitrogen), and anti-mycoticantibiotic (1:100; Invitrogen) in suspension in 6-well plates (Costar 3471; Corning, Corning, NY, USA).

### Animal model

Six-week-old male BALB/c nude mice were maintained under specific pathogen-free conditions in the Laboratory Animal Center of Sun Yat-sen University, and all experiments were performed according to our Institutional Animal Guidelines. Mice were randomly assigned to 2 groups (*N* = 6). In each group, lentiviral-transduced SK-Cha-1 cells (2.5 × 10^6^) were subcutaneously injected into the dorsal right flanks of the mice, and the mice were monitored every 3 days for tumor growth.

### Statistical analysis

The *t*-test was used to determine the significance between 2 groups. Data are expressed as the mean ± SEM of 3 independent experiments. One way analysis of variance was performed to compare multiple groups, and Dunnett’s test was used to analyze multiple comparisons. For clinical data analysis, the Kruskal-Wallis test was used when comparing multiple groups, and multiple comparisons were conducted using the least significant difference *t*-test. Spearman’s correction was used to determine the correlation between CTNNB1 expression with m6A writers and IGF2BP2 levels. Receiver operating characteristic (ROC) curves were used to determine the diagnostic utility of CTNNB1, m6A writers, and IGF2BP2. Discriminant analysis was used to identify the combination of molecules to build an optimal model of predicted probability. The sensitivity and specificity were obtained at the optimal cutoff points when the Youden’s index was maximal. *P* < 0.05 was considered statistically significant.

## Results

### The m6A modification writers were highly expressed in CCA and were regulated by IL-6 treatment

**Figure 1:**
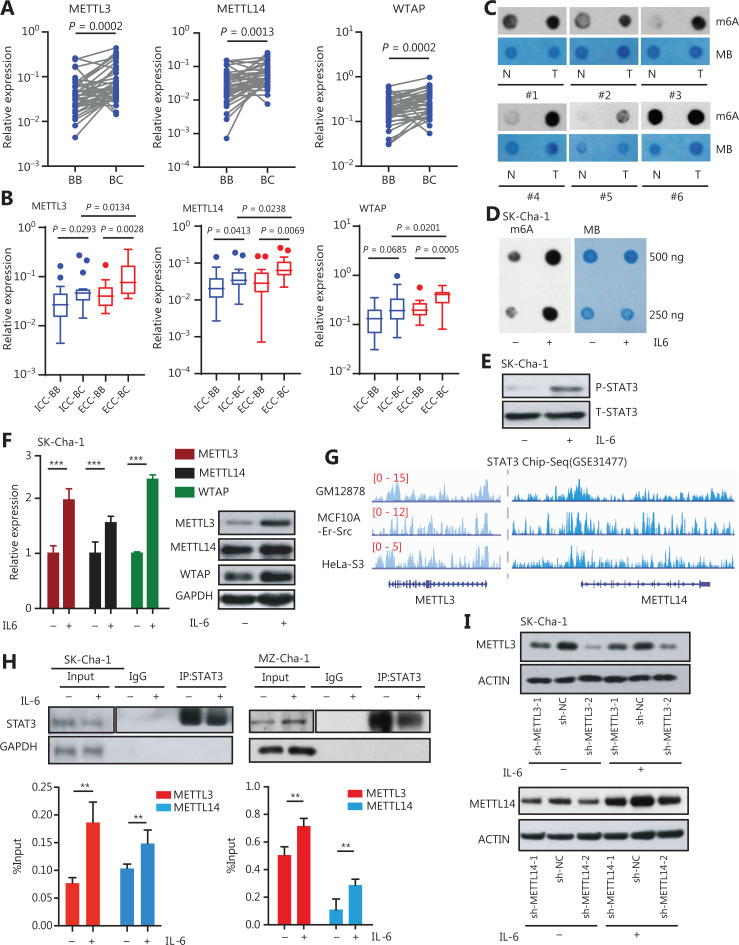
The m6A modification writers were highly expressed in CCAs and regulated by IL-6/STAT3 inflammatory signaling. (A) The m6A writers were more highly expressed in cancerous tissues than those in matched adjacent tissues of 38 paired CCA patient samples. The Wilcoxon matched-pairs signed rank test was used to calculate the significance. (B) The m6A writers were more highly expressed in extra-hepatic (ECC) cancerous tissues (*N* = 20) than in intra-hepatic (ICC) cancerous tissues (*N* = 18) from CCA patients. The m6A writers were also highly expressed in ICC and ECC samples compared to normal tissues. BB indicates CCA adjacent tissue, and BC indicates CCA cancerous tissue. (C) Dot blots showing the global m6A levels in matched pairs of cancerous and adjacent tissues from patients with CCA. MB indicates methylene blue, which shows the total RNA level. (D) Dot blots showing the global m6A levels between CCA cells treated with or without 20 ng/mL interleukin-6 (IL-6) for 2 h. MB indicates methylene blue. (E) Western blot analysis showed that the expression levels of phosphorylated STAT3 were significantly upregulated in CCA cells treated with 20 ng/mL IL-6 for 2 h. (F) The expression levels of METTL3, METTL14, and WTAP measured by qRT-PCR, and immunoblots of CCA cells treated with 20 ng/mL IL-6 for 2 h. Error bars denote ± SEM (***P < 0.001) in 3 independent experiments. (G) STAT3 was located at the genetic locus of METTL3 and METTL14 in the GES31477 data set. (H) The anti-STAT3 chromatin immunoprecipitation (ChIP) assay showed the enrichment of STAT3 located at the METTL3 and METTL14 gene loci in CCA cells treated with or without 20 ng/mL IL-6 for 2 h. Western blot for the STAT3 of ChIP/IP; glyceraldehyde 3-phosphate dehydrogenase served as the negative control. Error bars denote ± SEM (**P < 0.01) based on 3 independent experiments. (I) Western blot showed the expressions of METTL3 and METTL14 in the sh-METTL3 and sh-METTL14 CCA cells treated with or without 20 ng/mL IL-6 for 2 h, respectively.

To understand the function and regulatory mechanism of m6A modification in cancer cell stemness and inflammatory response in CCA development, we first detected the expressions of these m6A writers in 38 matched pairs of CCA cancerous and adjacent tissues. We found that m6A modification writers, including METTL3, METTL14, and Wilms’ tumor 1-associating protein (WTAP), were upregulated in the CCA tissues (**Figure 1A**), which was further validated by the CCA cohort in The Cancer Genome Atlas (TCGA) database (**Supplementary Figure S1**). Not only did the m6A writers show high expression levels in both ICC and ECC subgroups, but they also showed distinct expression patterns in these subgroups (**Figure 1B**). Because ICC and ECC subgroups had different clinical characteristics and manifestations, m6A writers could potentially be used to identify the liver metastatic foci of CCA cells and discriminate different CCA subtypes. The m6A dot blot assay showed that CCA tissue samples had a higher m6A level than the adjacent tissues (**Figure 1C**), suggesting that m6A modification and its writers highly expressed in CCA could be beneficial for CCA development.

Previous studies have shown that the inflammatory response plays critical roles in CCA development. We therefore hypothesized that the expression of writers may be enhanced by inflammatory responses in CCA cells. When CCA cells were exposed to IL-6, a key inflammatory factor in the CCA tumor microenvironment^8,26^, the level of the m6A modification was significantly elevated (**Figure 1D**), accompanied by higher expression levels of phosphorylated STAT3 in the CCA cells exposed to IL-6 (**Figure 1E**). Notably, IL-6 treatment significantly enhanced the expressions of MELLT3, METTL14, and WTAP in CCA cells (**Figure 1F**). These data suggested that the inflammatory response increased total RNA m6A levels and the expressions of m6A writers, leading to enhancement of CCA development. Mechanistically, we found that the expressions of m6A writers were regulated by the IL-6/STAT3 inflammatory pathway. Reanalyzing STAT3 ChIP-seq data (GES31477)^27^, we found a considerable enrichment of STAT3 at the METTL3, METTL14, and WTAP gene loci in the cell lines (**Figure 1G and Supplementary Figure S2**), indicating that IL-6/STAT3 inflammatory signaling, which regulated the m6A writers, may depend on the activation of STAT3. Subsequently, we performed ChIP assays using anti-STAT3 antibody in CCA cell lines, which showed that the STAT3 ChIP assays showed significant enrichment of STAT3 located at the METTL3 and METTL14 gene loci in CCA cells (**Figure 1H**). As shown in **Figure 1I and Supplementary Figure S3A, S3B**, knockdown of METTL3 or METTL14 also reduced the increased expression levels of m6A writers triggered by IL-6 treatment. Together, these results showed that m6A modification writers were highly expressed in CCA and were direct downstream genes of the IL-6/STAT3 inflammatory pathway.

### The m6A writers promoted CCA cell stemness in inflammatory responses

**Figure 2:**
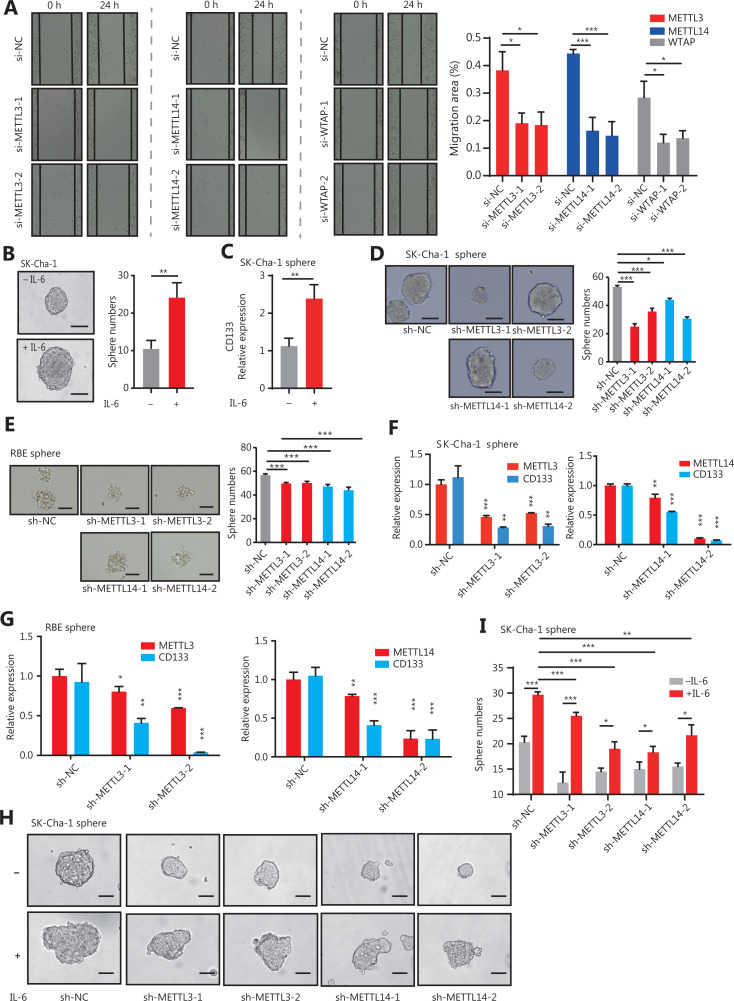
The m6A writers maintained the stem-like properties of CCA cells in response to inflammation. (A) Using a wound healing assay, the cell motilities of METTL3, METTL14, and WTAP-silenced CCA SK-Cha-1 cells were observed at 0 and 24 h following wounding by a pipette tip. Original magnification: 100×. Quantitative analysis of the migration. Error bars denote ± SEM (*P < 0.05; ***P < 0.001) based on 3 independent experiments. (B) Morphology and number of mammospheres of CCA cells treated with or without 20 ng/mL IL-6 for 2 h. Scale bars: 50 μm. Error bars denote ± SEM (*P < 0.05; ***P < 0.001). (C) The qRT-PCR showing the expression levels of CD133 in SK-Cha-1 cell mammospheres in the IL-6 treatment *vs*. control groups. Error bars denote ± SEM (***P < 0.001) based on 3 independent experiments. (D, E) Morphology and numbers of mammospheres of SK-Cha-1 cells upon sh-METTL3 (D) and METTL14 (E) knockdown. Scale bars: 50 μm. Error bars denote ± SEM (*P < 0.05; **P < 0.01; ***P < 0.001) in 3 independent experiments. (F, G) The qRT-PCR showed the expression levels of METT3, METTL14, and CD133 significantly downregulated in the knockdown of METTL3 (F) and METTL14 (G) SK-Cha-1 cell mammospheres. (H) Measurement of the morphology and number of mammospheres after 20 ng/mL IL-6 treatment in sh-METTL3 or sh-METTL14 CCA cells for 2 h, respectively. Scale bars: 50 μm. Error bars denote ± SEM (*P < 0.05; ***P < 0.001) in 3 independent experiments. (I) The qRT-PCR showed the expression levels of CD133 after 20 ng/mL IL-6 treatment in the sh-METTL3 or sh-METTL14 CCA cells for 2 h. Error bars denote ± SEM (*P < 0.05; ***P < 0.001) in 3 independent experiments.

Previous studies have shown that the activation of IL-6/STAT3 signaling is an important contributor to CCA^8,28,29^. We next investigated the function of m6A writers, involving the direct downstream genes of the IL-6/STAT3 inflammatory signaling, on the pathogenesis of CCA in sh-METTL3, sh-METTL14, and sh-WTAP cells (**Supplementary Figure S3A**). The CCK-8 and scratch wound healing assays showed that silencing m6A writers dramatically prevented the proliferation and migration of the CCA cells (**Figure 2A and Supplementary Figure S3C**), suggesting their tumor-promoting roles. We also showed that IL-6 treatment regulated the stem-like properties of CCA cells (**Figure 2B, 2C**), indicating a potential role of m6A writers in the stemness of CCA cells. To determine whether METTL3, METTL14, and WTAP regulated CCA cell stemness induced by inflammatory responses, we further performed mammosphere culture assays. Silencing of m6A genes in CCA cells resulted in a significant reduction in mammosphere numbers (**Figure 2D, 2E**). Consistently, the expression levels of CD133, a marker of CCA stem cells, were also decreased in mammospheres with downregulated METTL3 and METTL14 (**Figure 2F, 2G**). More importantly, knockdown of METTL3 or METTL14 extensively reduced the increase of CCA stem-like properties, including the number of mammospheres and the expression of CD133 in mammospheres, which were triggered by IL-6 treatment in CCA cells (**Figure 2H, 2I**). Together, these results showed that inflammatory responses controlled the stem-like properties of CCA cells through m6A modification triggered by METTL3, METTL14, and WTAP genes.

### The m6A writers maintained the expression of stemness-related genes through m6A modification during inflammation

**Figure 3:**
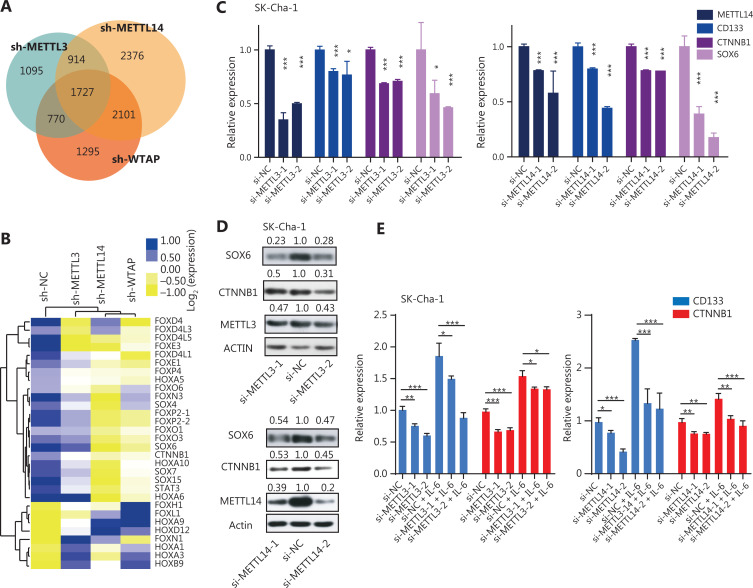
The m6A writers maintained the expression of stemness-related genes in inflammatory responses. (A) Venn diagram for the aberrant genes in the sh-METTL3, sh-METTL14, and sh-WTAP samples. (B) Heat map for the top 30 differentially-expressed stemness-related genes in m6A gene knocked-down cells. (C) The qRT-PCR showing the expression levels of METTL3, METTL14, CD133, CTNNB1, and SOX6 in METTL3 or METTL14-silenced CCAs. Error bars denote ± SEM (*P < 0.05; ***P < 0.001) in 3 independent experiments. (D) Knockdown of METTL3 or METTL14 reduced the CTNNB1 and SOX6 protein levels. The METTL3/ACTIN, METTL14/ACTIN, CTNNB1/ACTIN, or SOX6/ACTIN densitometric ratio was recorded using ImageJ software. (E) The qRT-PCR showing the expression levels of CD133 and CTNNB1 in the m6A writer knockdown cells (with or without 20 ng/mL IL-6 exposure). Error bars denote ± SEM (*P < 0.05; **P < 0.01; ***P < 0.001) in 3 independent experiments.

**Figure 4:**
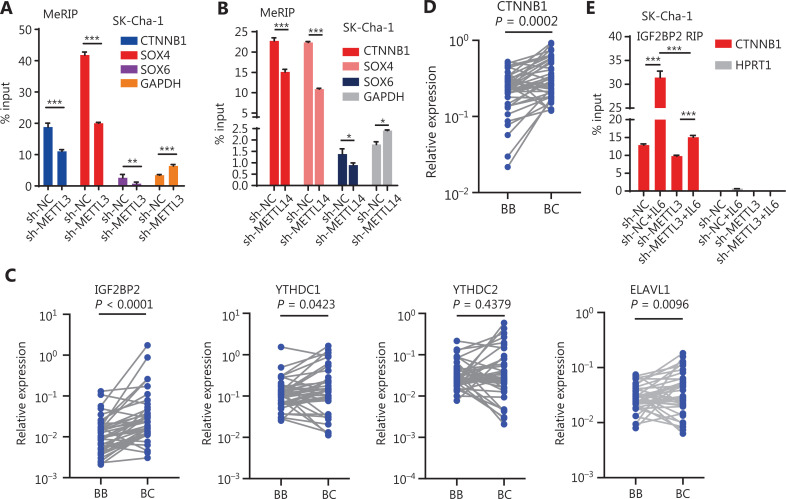
The m6A writers regulate the expression of stemness-related genes in a m6A depended manner. (A, B) METTL3 (A) and METTL14 (B) knockdown reduced the enrichment of m6A modifications of CTNNB1, SOX4, and SXO6. Error bars denote ± SEM (*P < 0.05; **P < 0.01; ***P < 0.001) in 3 independent experiments. (C, D) IGF2BP2 (5.9-fold changes; *P* < 0.0001), YTHDC1 (1.7-fold changes; *P* = 0.0423), ELAVL1 (1.6-fold changes; *P* = 0.0096), and YTHDC2 (*P* = 0.4379) (C), and CTNNB1 (*P* = 0.0002) (D) are highly expressed in cancerous tissues compared to those in matched adjacent tissues of 38 paired CCA patient samples. Wilcoxon matched-pairs signed rank test was used to calculate the significance. BB indicates CCA adjacent tissue, and BC indicates CCA cancerous tissue. (E) The enrichment of IGF2BP2 on CTNNB1 mRNA in the METTL3 knockdown cells treated with or without IL-6. Error bars denote ± SEM (***P < 0.001) in 3 independent experiments, using HPRT1 as the negative control.

To determine whether these inflammatory responses controlled the stem-like properties of CCA cells through m6A modification, we used RNA-seq to screen stemness-related genes that may have been regulated by m6A writers. The results showed that METTL3, METTL14, and WTAP shared 1727 RNA targets (**Figure 3A and Supplementary Figure S4A**). Notably, a set of stemness genes, such as SOX4, SOX6, and CTNNB1, was significantly downregulated in cells when m6A writers were knocked down (**Figure 3B-D and Supplementary Figure S4B**). Additionally, knockdown of METTL3 or METTL14 reduced the increased expression levels of CD133 and CTNNB1 triggered by IL-6 treatment, suggesting that the inflammatory response maintained the stem-like properties of CCA cells through modification of m6A modification (**Figure 3E**). It is well-known that m6A modification is the functional mechanism by which m6A writers affect target genes^11,13,30^. Thus, we next investigated whether METTL3/14 regulated the expression of stemness genes in an m6A modification manner. An m6A RNA immunoprecipitation assay showed that the lower enrichment of m6A modification was associated with some stemness-regulated genes, including CTNNB1, in METTL3- and METTL14-silenced CCA cells, which is consistent with the data in the GSE90642 data set based on a study of HepG2 cells^31^ (**Figure 4A, B and Supplementary Figure S5A**). Together, these findings suggested that m6A writers regulated the expression of stemness-related genes through an m6A-dependent manner in response to inflammation.

### The m6A writers regulated the mRNA stability and translation of stemness-related genes *via* m6A modification during inflammation

**Figure 5:**
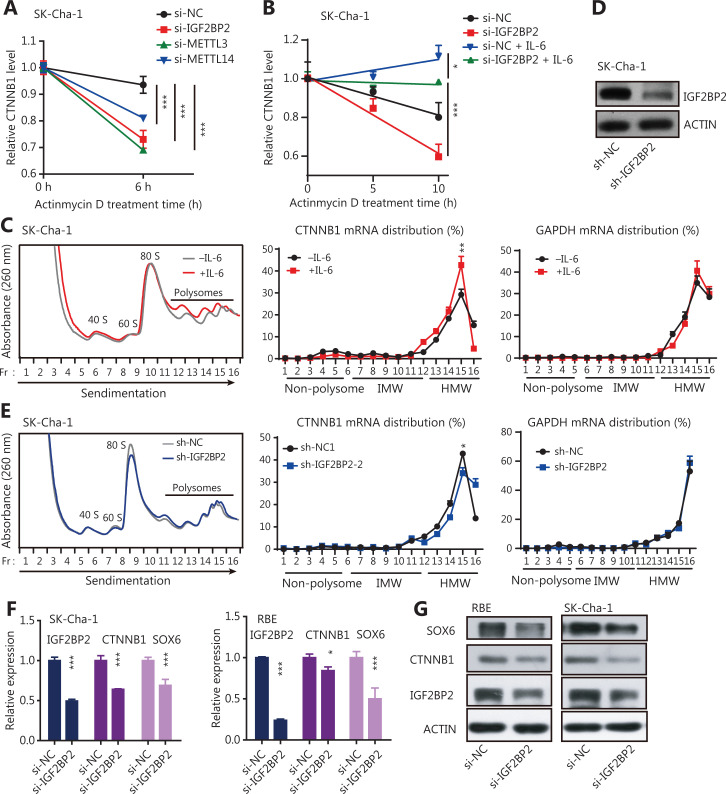
The m6A writers maintained the expression of stemness-related genes in a m6A-IGF2BP2-dependent manner in inflammatory responses. (A) RNA stability of CTNNB1 transcripts detected by qRT-PCR in IGF2BP2, METT3, or METTL14 knockdown CCA cells. Actinomycin D (Act D) has been used in the assays. Error bars denote ± SEM (***P < 0.001) in 3 independent experiments. (B) The RNA stability of CTNNB1 transcripts was detected by qRT-PCR in IGF2BP2 knockdown SK-Cha-1 cells (with or without 20 ng/mL IL-6 treatment for 2 h). The transcription inhibitor, Act D has been used in the assays. Error bars denote ± SEM (*P < 0.05; ***P < 0.001) in 3 independent experiments. (C) Polysomes in extracts of SK-Cha-1 cells treated for 2 h with or without 20 ng/mL IL-6 was fractionated using sucrose gradients, and the relative levels of CTNNB1 mRNA were analyzed by qRT-PCR in the gradient fractions. Glyceraldehye 3-phosphate dehydrogenase (GAPDH) mRNA was used as the negative control. The enrichment of the CTNBB1 mRNA at the peak point in the HMW fraction was significantly increased after IL-6 treatment (**P < 0.01). (D) Immunoblots showing the expression levels of IGF2BP2 in sh-IGF2BP2-downregulated SK-Cha-1 cells. (E) Polysome profiling of CTNNB1 mRNA in sh-NC and sh-IGF2BP2 SK-Cha-1 cells. GAPDH mRNA acted as the negative control. The enrichment of the CTNBB1 mRNA at the peak point in the HMW fraction was significantly decreased after IGF2BP2 knockdown (*P < 0.05). (F) The qRT-PCR showing the expression levels of IGF2BP2, CTNNB1, and SOX6 in IGF2BP2-downregulated CCA cells. Error bars denote ± SEM (*P < 0.05; ***P < 0.001) in 3 independent experiments. (G) Immunoblots showing the expression levels of CTNNB1 and SOX6 in si-IGF2BP2 CCA cells.

It was reported that m6A affected mRNA stability and translation, which was mediated by specific m6A-binding proteins known as m6A readers^30–32^. To search for specific m6A readers in CCA cells, we again analyzed TCGA database and found that 6 m6A readers, including IGF2BP2, YTH domain containing 2 (YTHDC2), embryonic lethal, abnormal vision, and Drosophila-like protein 1 (ELAVL1), were highly expressed in CCA tumors, with IGF2BP2 displaying the highest expression (**Supplementary Figure S5B**). We also confirmed the expression patterns of these m6A readers in 38 paired CCA samples and found that the expression of IGF2BP2 was the highest (5.9-fold change; *P* < 0.0001) (**Figure 4C**). Notably, many stemness-related RNAs, including CTNNB1, were significantly enriched with IGF2BP2 in HepG2 cells (**Supplementary Figure S5C**). These results suggested that the highly expressed IGF2BP2 might have a critical role in CCA progression, and might directly control the stability and translation of stemness-related transcripts in an m6A-dependent manner. To validate this possibility, we measured the enrichment of IGF2BP2 on CTNNB1 mRNA, which was highly expressed in CCA tumors (**Figure 4D**). The results showed that the enrichment of IGF2BP2 significantly increased after IL-6 treatment; however, the increase of IGF2BP2 enrichment by IL-6 was reduced by knockdown of METTL3 (**Figure 4E**). We also treated CCA cells with the transcription inhibitor, actinomycin D (Act D)^25,31^, and then determined the RNA stability of CTNNB1 transcripts in the control and METTL3-, METTL14-, or IGF2BP2-knockdown cells (**Supplementary Figure S3A, S5D**). Knocking down METTL3, METTL14, or IGF2BP2 resulted in a significant increase in the degradation of CTNNB1 (**Figure 5A**), and the degradation of CTNNB1 caused by IGF2BP2 knockdown was reversed by IL-6 treatment (**Figure 5B**). These findings showed that the higher m6A modification of CTNNB1 transcripts was induced by the IL-6/STAT3 inflammatory pathway, and was recognized by IGF2BP2, a special m6A reader, which regulated CTNNB1 mRNA stability.

To investigate whether IGF2BP2 regulated the translational efficiency of CTNNB1, a process that is dependent on mRNA stability, polysome profile experiments were conducted. In principle, mRNAs distributed to non-polysomal fractions were inefficiently translated, with low molecular weight fractions having a moderate translational efficiency; and high molecular weight (HMW) fractions having a high translational efficiency^33,34^. IL-6 treatment of the CCA cell line, SK-Cha-1, caused a greater enrichment of the CTNNB1 mRNA in the HMW portion, whereas it did not change the polysome distribution profile of GAPDH mRNA (**Figure 5C**). This indicated that increased m6A modification of the CTNNB1 transcripts induced by the IL-6/STAT3 inflammatory pathway enhanced their translational efficiencies. In addition, knockdown of IGF2BP2 in SK-Cha-1 cells significantly decreased the enrichment of CTNNB1 mRNA in the HMW fraction, with GAPDH mRNA acting as a negative control (**Figure 5D, 5E**), suggesting that IGF2BP2 might increase the translational efficiency of CTNNB1 mRNA in an m6A-dependent manner. The expression levels of stemness-related genes were also significantly reduced in IGF2BP2-knockdown CCA cells (**Figure 5F, 5G**). Overall, these results showed that the inflammatory response facilitated cancer cell stemness was modulated by the stability and translation of stemness-related transcripts in an m6A-IGF2BP2-dependent manner in CCA cells.

### The m6A writers maintained CCA cell stemness *in vivo* and their clinical relevance in CCA

**Figure 6:**
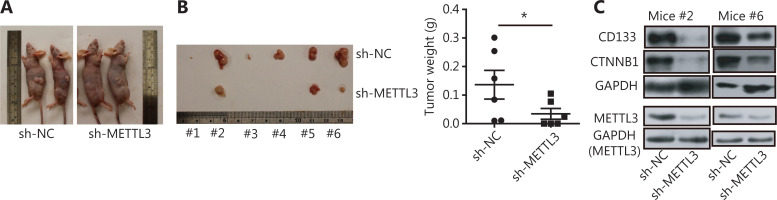
METTL3 maintained CCA cell stemness *in vivo* and facilitated tumor progression. (A) Representative images for the tumors of implantations of the shRNA-transformed SK-Cha-1 cells into the flanks of 6-week-old male nude mice. (B) The knockdown of METTL3 inhibited malignant proliferation of SK-Cha-1 cells and tumor size and weight *in vivo*. Error bars denote ± SEM (6 mice per group; *P < 0.05). (C) Immunoblots showing the expression levels of CTNNB1, CD133, and METTL3 in tumors from METTL3 knockdown mice.

**Figure 7:**
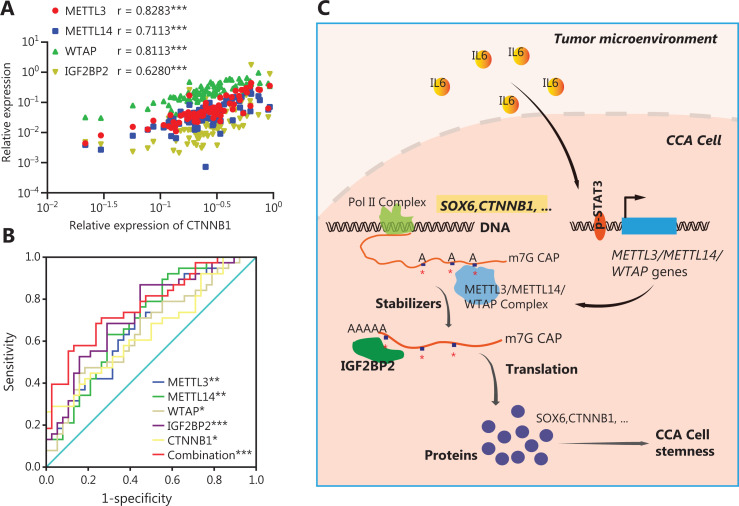
Clinical relevance of m6A writers in CCA and schematic representations of pathways linking m6A, IL-6, and CCA cell stemness. (A) CTNNB1 expression positively correlated with both m6A writers and IGF2BP2 in 38 paired CCA tumor and adjacent normal tissues. Spearman’s correction was used to analyze the significance (***P < 0.001). (B) Diagnostic value of m6A writers and IGF2BP2, and CTNNB1 for CCA. The areas under the curve were 0.6794 for METTL3 [95% confidence interval (CI): 0.5597–0.7990, *P* < 0.01], 0.6994 for METTL14 (95% CI: 0.5816–0.8172; *P* < 0.01), 0.6524 for WTAP (95% CI: 0.5291–0.7756; *P* < 0.05), 0.7313 for IGF2BP2 (95% CI: 0.6183–0.8443; *P* < 0.001), 0.6496 for CTNNB1 (95% CI: 0.5264–0.7728; *P* < 0.05), and 0.7722 for the combination (95% CI: 0.6679–0.8764; *P* < 0.001). The sensitivity and specificity at each cutoff point were as follows: 86.84% and 44.74% for METTL3, 92.11% and 42.11% for METTL14, 47.37% and 81.58 % for WTAP, 86.84% and 55.26 % for IGF2BP2, 28.95% and 97.37% for CTNNB1, and 68.42 % and 76.32% for the combination. (C) A working model for the function of m6A writers in facilitating cancer cell stemness in CCA. In the tumor microenvironment, the inflammatory factor, IL-6, can trigger the STAT3 pathway. The activated STAT3 located at the METTL3, METTL14, and WTAP gene loci then increased their expressions. This increases the expression of m6A genes, which contributes to an increase in m6A methylation of stemness-related transcripts, including SOX6 and CTNNB1. Methylated stemness-related transcripts are subsequently recognized by IGF2BP2, to maintain their mRNA stability and expression, resulting in abnormal CCA cell stemness and pathogenesis of CCA.

Using a subcutaneous xenotransplantation model, we determined whether m6A genes promoted CCA cell stemness and development^8^. METTL3 is the major m6A modification enzyme that has a core role in enhancing stem-like properties in various types of cancers^16,17,20,21^. We have also shown that METTL3 showed powerful effects on stem-like properties of CCA cells *via* m6A modification. Thus, METTL3 was selected to perform the *in vivo* experiments in this study. When METTL3 knockdown SK-Cha-1 cells were implanted, tumor growth was inhibited, and the xenograft tumor weight was reduced, compared to controls (**Figure 6A, 6B**). In addition, the expression levels of CD133 and CTNNB1 were significantly decreased in tumors from mice implanted with METTL3 knockdown CCA cells (**Figure 6C**). Based on the mechanism and function of m6A writers in CCA cells identified *in vitro* and *in vivo*, we determined the clinical relevance of m6A writers, IGF2BP2, and CTNNB1. CTNNB1 expression positively correlated with both m6A writers and IGF2BP2 in 38 paired CCA tumor and adjacent normal tissues (**Figure 7A**), which was further validated by data sets in TCGA database (**Supplementary Figure S6**). Moreover, ROC curve analysis was performed with the relative expression of m6A writers, IGF2BP2, and CTNNB1, and the associated area under the ROC curve (AUC), as well as the sensitivity and specificity being used to evaluate the diagnostic potency in CCA. **Figure 7B** shows that all 5 molecules showed considerable AUC values and significantly distinguished the CCA tumors from adjacent normal tissues. Notably, the combination of m6A writers, IGF2BP2 and CTNNB1, showed an additive predictive value, and the AUC was up to 0.7722 (95% CI: 0.6679 to 0.8764; *P* < 0.001) with a cutoff point of 68.42% sensitivity and 76.32% specificity (**Figure 7B**). These results suggested that changes in the levels of m6A writers, IGF2BP2, and CTNNB1, were potential tools for the detection of CCA, especially when these molecules were simultaneously evaluated.

Taken together, our data showed that the inflammatory response triggered by IL-6 enhanced the expressions of m6A genes by activating the STAT3 pathway, and then regulated the stability and translation of stemness-related genes in an m6A-IGF2BP2-dependent manner, ultimately facilitating CCA cell stemness and tumor progression (**Figure 7C**). This is the first report showing that m6A modification was linked with the inflammatory response to cell stemness in CCA.

## Discussion

Cancer cell stemness is reported to be critical for cancer initiation, metastasis, relapse, and chemoresistance^1,35–38^. Investigation of the regulatory mechanisms of CSCs in CCA is essential for developing effective therapies that improve patient outcomes^1,36,37^. Previous studies have indicated that aberrant inflammatory factors, including IL-6, are increased in patients with CCA, induce inflammatory responses, and are closely associated with stemness maintenance in CCA^29,36,39,40^. The m6A modification plays important roles in cell stemness and tumorigenesis^17,19–21^. However, whether m6A modification is involved in the inflammatory response and cell self-renewal in CCA remains unknown.

Using loss-of-function experiments, we showed that the inflammatory response triggered by IL-6 facilitated cancer cell stemness and tumor progression in an m6A-IGF2BP2-dependent manner in CCA patients. The results showed that the expressions of m6A writers in CCA cells were upregulated by IL-6. Mechanistically, IL-6/STAT3 signaling improved the expressions of m6A writers in gene regions, which directly bound to activated STAT3. The m6A RNA modification can significantly enhance the stability and translation of stem genes, including CTNNB1^41^ and CD133^8^, and facilitate the stem-like properties of CCA, highlighting its crucial role in the self-renewal of CCA cells and its potential as a valuable therapeutic target for CCA treatment. Clinically, we have also shown that m6A writers, IGF2BP2 and CTNNB1, were highly expressed in CCA patient samples, suggesting their potential roles for the detection of CCA, especially when these molecular were simultaneously evaluated. Of note, m6A writers presented distinct expression patterns in ICC and ECC samples, implying that they could potentially identify different CCA subtypes and may contribute to their differences in etiologies and pathogenesis. The study has also provided insight into the inflammatory response that regulates CCA stemness gene expression through an m6A-dependent pathway.

Recent progress has indicated that m6A plays indispensable roles in inflammation and antitumor effects through its interactions with various m6A regulators, such as IGF2BPs, YTH domain containing 1(YTHDC1), etc.^12,30,32^. Most studies have focused on m6A as a regulator of inflammatory signaling^42–44^. For example, YTH N(6)-methyl adenosine RNA binding protein 2 (YTHDF2) serves as a tumor suppressor to resolve cancer-promoting inflammation by degrading interleukin 11 mRNAs in HCCs^42^, and METTL3 depletion decreased the expressions of inflammatory cytokines in human dental pulp cells^43^. Different from these reports, our results showed that the inflammatory pathway can regulate m6A modification by directly binding to the gene loci of m6A writers to activate the transcription process of m6A methylase to increase total m6A levels in CCA cells. These findings identified the upstream regulatory mechanism of the m6A modification and highlighted the important roles of the inflammatory pathway in the regulation of epigenetic alterations.

Increased evidence has led to an appreciation of the connection between cancer cell stemness maintenance and m6A modification, where a universal regulation model functions as a signal to mark specific RNAs, whose fate is determined by the “reader” protein that subsequently recognizes and interacts with it^17,19,45^. It seems that 1 type of cancer involves having a main m6A reader that recognizes the m6A modification and regulates cancer cell stemness^21,46^. For example, YTHDF2 is highly expressed in hepatocellular carcinoma and specifically reads the m6A modification of OCT4, and enhances its expression, which promotes the liver cancer stem cell phenotype and cancer metastasis^46^. For colorectal carcinoma cells, highly expressed IGF2BP2 can regulate sex-determining region Y-box 2 (SOX2) expression through an m6A-IGF2BP2-dependent mechanism to maintain cell self-renewal^21^. In this study, we showed that IGF2BP2 displayed the highest expression pattern in CCA cells, and specifically promoted CTNNB1 expression in an m6A-dependent manner, indicating that it may be a key m6A reader in CCA patients. Furthermore, our study screened a set of cancer cell genes in different stemness-related signaling pathways, including CTNNB1, SOX6, and CD133 in CCA, to show that stemness genes were targeted by m6A modification. We found that the m6A/IGF2BP2 axis functioned in maintaining CCA stemness, suggesting that it might be a new target for CCA diagnosis and treatment.

## Conclusions

Collectively, we showed that m6A writers were highly expressed in CCA, and could potentially be used as diagnostic biomarkers for CCA. In CCA patients, the inflammatory factors regulating cancer cell stemness and tumor progression may be partially dependent on m6A modifications. IL-6/STAT3 signaling can facilitate the expression of m6A genes, which enhances m6A modifications related to stemness-related genes, to improve their mRNA stabilities and translations in an m6A-IGF2BP2-dependent manner. This study therefore provided insight into the mechanisms of the inflammatory response and CCA cell stemness, and suggested that m6A modification is a targetable mediator in the response to exposure to inflammatory factors.
