# Supplementary material for: The m6A writers regulated by the IL-6/STAT3 inflammatory pathway facilitate cancer cell stemness in cholangiocarcinoma
Source: Cancer Biol Med. 2021 Aug 4;19(3):343–57. doi: 10.20892/j.issn.2095-3941.2020.0661 (PMC8958887; doi:10.20892/j.issn.2095-3941.2020.0661)
Supplement: Supplementary file 1 [file cbm-19-343-s001.pdf]

Supplementary materials

Table S1 Patient demographics and clinicopathological features

Characteristics	Intra-hepatic CCA	Extra-hepatic CCA
Number	*N* = 18 (52.0%)	*N* = 20 (48.0%)
Age (years, median and range)	61 (45–79)	59 (33–86)
Sex (%)		
Male	10 (55.6)	11 (55.0)
Female	8 (44.4)	9 (45.0)
HBV+ (%)	9 (50.0)	2 (10.0)
Tumor size (cm, median and range)	5.0 (1.2–12.0)	2.7 (1.2–5.0)
CA19-9 (U/mL, median and range)	213.2 (0.6–17929.9)	160.6 (0.69–3925)
Differentiation stage (%)		
Well	5 (27.7)	6 (30.0)
Moderately	6 (33.3)	7 (35.0)
Moderately-poorly	1 (5.6)	0 (0.0)
Poorly	3 (16.7)	4 (20.0)
N/A[a]	3 (16.7)	3 (15.0)

[a]Not applicable.

Table S2 Sequences for primers, siRNAs, and shRNAs used in this study

Primers	Sequences (5′ → 3′)
qPCR primers	
qMETTL3-forward	TTGTCTCCAACCTTCCGTAGTG
qMETTL3-reverse	CCAGATCAGAGAGGTGGTGTAG
qMETTL14-forward	AGAAACTTGCAGGGCTTCCTAT
qMETTL14-reverse	TCTTCTTCATATGGCAAATTTTCT
qWTAP-forward	CCTCTTCCCAAGAAGGTTCGATT
qWTAP-reverse	TGCAGACTCCTGCTGTTGTTG
qGAPDH-forward	GTCTCCTCTGACTTCAACAGCG
qGAPDH-reverse	ACCACCCTGTTGCTGTAGCCAA
qIGF2BP2-forward	GTTGGTGCCATCATCGGAAAGG
qIGF2BP2-reverse	TGGATGGTGACAGGCTTCTCTG
qCTNNB1-forward	CATCTACACAGTTTGATGCTGCT
qCTNNB1-reverse	GCAGTTTTGTCAGTTCAGGGA
qSOX4-forward	GCACTAGGACGTCTGCCTTT
qSOX4-reverse	ACACGGCATATTGCACAGGA

Table S2 Continued

Primers	Sequences (5′ → 3′)
qSOX6-forward	CAACACCAGGAAGAGACTTACGA
qSOX6-reverse	TCAAAGCGCGTTCTTTCCTTT
qCD133-forward	AGTCGGAAACTGGCAGATAGC
qCD133-reverse	GGTAGTGTTGTACTGGGCCAAT
qNanog-forward	AAGGTCCCGGTCAAGAAACAG
qNanog-reverse	CTTCTGCGTCACACCATTGC
qHPRT1-forward	CATTATGCTGAGGATTTGGAAAGG
qHPRT1-reverse	CTTGAGCACACAGAGGGCTAC
ChIP primers for qPCR	
ChIP-METTL3-forward	GAGAGAGCGGGTATGAGGACTAT
ChIP-METTL3-reverse	GCTGCTTCTTGTGGGCCTGGAT
ChIP-METTL14-forward	GATGAGATTGCAGCACCTCGATCA
ChIP-METTL14-reverse	AGCACCATCTTACCACTCTTCCAA
meRIP primers for qPCR	
meRIP-CTNNB1-forward	TCTGAACTTGCATTGTGATTGGC
meRIP-CTNNB1-reverse	GTCGTTTCCGCAACAAGTCC
meRIP-SOX6-forward	GGCAGCAAATGGACCTTG
meRIP-SOX6-reverse	GGGAAGAGGAATCCCTGTTGG
meRIP-SOX4-forward	AGTTTTCTAGAGACTTGAAGGAGTCTCC
meRIP-SOX4-reverse	CCTGCGTGGAGTCTCTCAGT
SiRNA/shRNA	
Si-METTL3-1-S	5′CUGCAAGUAUGUUCACUAUGA dTdT 3′
Si-METTL3-1-AS	3′dTdT GACGUUCAUACAAGUGAUACU 5′
Si-METTL3-2-S	5′AGGAGCCAGCCAAGAAAUCAA dTdT 3′
Si-METTL3-2-AS	3′dTdT UCCUCGGUCGGUUCUUUAGUU 5′
Si-METTL14-1-S	5′AAGGAUGAGUUAAUAGCUAAA dTdT 3′
Si-METTL14-1-AS	3′dTdT UUCCUACUCAAUUAUCGAUUU 5′
Si-METTL14-2-S	5′UGGUGCCGUGUUAAAUAGCAA dTdT 3′
Si-METTL14-2-AS	3′ dTdT ACCACGGCACAAUUUAUCGUU 5′
Si-WTAP-1-S	5′AAGGUUCGAUUGAGUGAAACA dTdT 3′
Si-WTAP-1-AS	3′ dTdT UUCCAAGCUAACUCACUUUGU 5′
Si-WTAP-2-S	5′GGGCAAGUACACAGAUCUUAA dTdT 3′
Si-WTAP-2-AS	3′ dTdT CCCGUUCAUGUGUCUAGAAUU 5′
Si-IGF2BP2-S	5′GCAAUUUAUACGAGGUUGU dTdT 3′
Si-IGF2BP2-AS	3′ dTdT CGUUAAAUAUGCUCCAACA 5′

Table S2 Continued

Primers	Sequences (5′ → 3′)
Sh-METTL3-1-S	5′-GATCCCTGCAAGTATGTTCACTATGACTTCCTGTCAGATCATAGTGAACATACTTGCAG TTTTTG-3′
Sh-METTL3-1-AS	5′-AATTCAAAAACTGCAAGTATGTTCACTATGATCTGACAGGAAGTCATAGTGAACATACTTGCAGG-3′
Sh-METTL3-2-S	5′-GATCCAGGAGCCAGCCAAGAAATCAACTTCCTGTCAGATTGATTTCTTGGCTGGCTCCTTTTTTG-3′
Sh-METTL3-2-S	5′-AATTCAAAAAAGGAGCCAGCCAAGAAATCAATCTGACAGGAAGTTGATTTCTTGGCTGGCTCCTG-3′
Sh-METTL14-1-S	5′-GATCCAAGGATGAGTTAATAGCTAAACTTCCTGTCAGA TTTAGCTATTAACTCATCCTT TTTTTG-3′
Sh-METTL14-1-AS	5′-AATTCAAAAAAAGGATGAGTTAATAGCTAAATCTGACAGGAAGTTTAGCTATTAACTCATCCTT G-3′
Sh-METTL14-2-S	5′-GATCCTGGTGCCGTGTTAAATAGCAACTTCCTGTCAGATTGCTATTTAACACGGCACCA TTTTTG-3′
Sh-METTL14-2-AS	5′-AATTCAAAAATGGTGCCGTGTTAAATAGCAATCTGACAGGAAGTTGCTATTTAACACGGCACCAG-3′
Sh-WTAP-1-S	5′-GATCCAAGGTTCGATTGAGTGAAACACTTCCTGTCAGATGTTTCACTCAATCGAACCTT TTTTTG-3′
Sh-WTAP-1-AS	5′-AATTCAAAAAAAGGTTCGATTGAGTGAAACATCTGACAGGAAGTGTTTCACTCAATCGAACCTT G-3′
Sh-WTAP-2-S	5′-GATCCGGGCAAGTACACAGATCTTAACTTCCTGTCAGATTAAGATCTGTGTACTTGCCC TTTTTG-3′
Sh-WTAP-2-AS	5′-AATTCAAAAAGGGCAAGTACACAGATCTTAATCTGACAGGAAGTTAAGATCTGTGTACTTGCCC G-3′
Sh-IGF2BP2-S	5′-GATCCGCAATTTATACGAGGTTGTCTTCCTGTCAGAACAACCTCGTATAAATTGC TTTTTG-3′
Sh-IGF2BP2-AS	5′-AATTCAAAAAGCAATTTATACGAGGTTGTTCTGACAGGAAGACAACCTCGTATAAATTGC G-3′

Table S3 All of the antibodies and reagents used in this study

Name	Species	Company	NO.
CD133	Rabbit	Proteintech	18470-1-AP
P-STAT3	Rabbit	CST	9145S
T-STAT3	Rabbit	Santa Cruz	sc-8019
METTL3	Rabbit	Proteintech	15073-1-AP
METTL14	Rabbit	Proteintech	26158-1-AP
WTAP	Mouse	Proteintech	60188-1-Ig
CTNNB1	Rabbit	CST	8480S
GAPDH polyAb	Rabbit	Proteintech	10494-1-AP
β-actin	Mouse	Sigma-Aldrich	A2228
Goat Anti-Mouse IgG HRP	Mouse	Thermo Fisher	H10007
Goat Anti-Rabbit IgG HRP	Rabbit	Thermo Fisher	A18903
m6A	Rabbit	Synaptic System	202003
IGF2BP2	Mouse	Santa Cruz	sc-377014

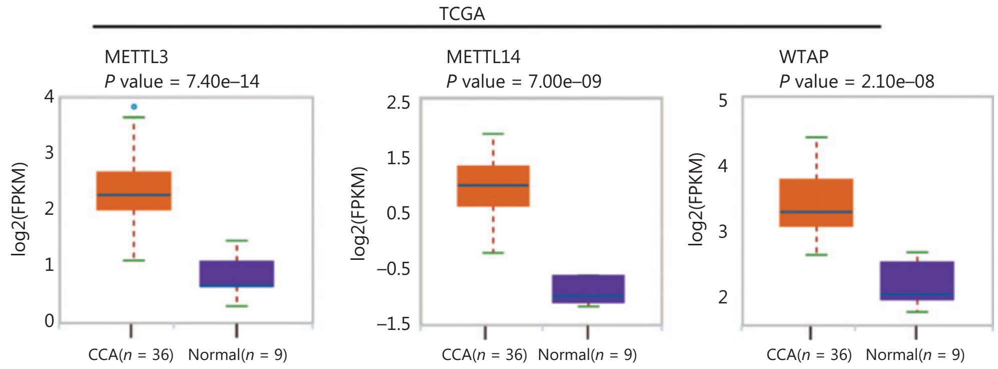

Figure S1 Expression of m6A genes in The Tumor Cancer Genome Atlas (TCGA) database. The expressions of METTL3, METTL14, and WTAP in TCGA database of cholangiocarcinomas.

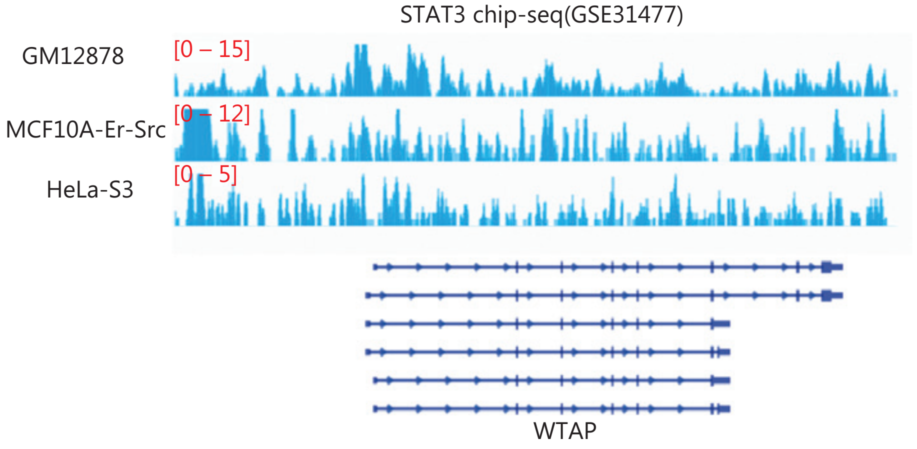

Figure S2 STAT3 was located at the genetic locus of *WTAP* in the GES31477 data set.

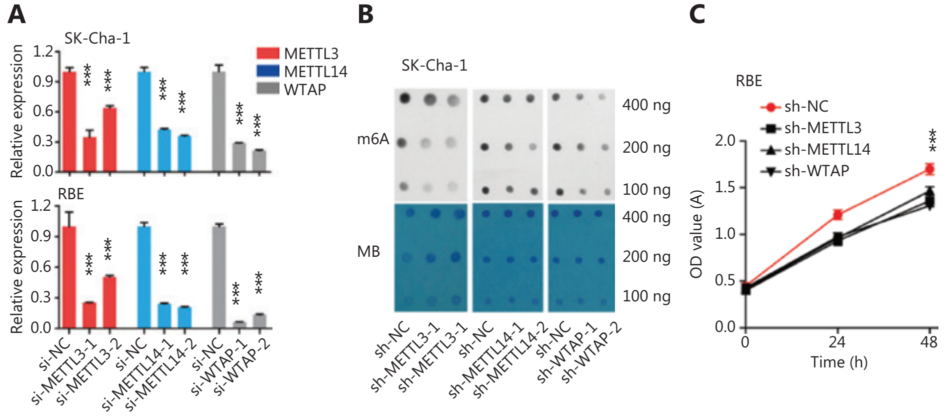

Figure S3 Knocking down m6A writers inhibited cholangiocarcinoma (CCA) cell proliferation. (A) The qRT-PCR showing the knockdown efficiency of METTL3, METTL14, and WTAP in CCA cells. Error bars denote ± SEM (*** $P < 0.001$) in 3 independent experiments. (B) Dot blots showing the global m6A levels after knocking-down m6A genes. (C) Cell proliferation was measured using a CCK-8 assay at 0, 24, and 48 h in METTL3-, METTL14-, and WTAP-silenced CCA cells. Error bars denote ± SEM (***$P < 0.001$) in 3 independent experiments.

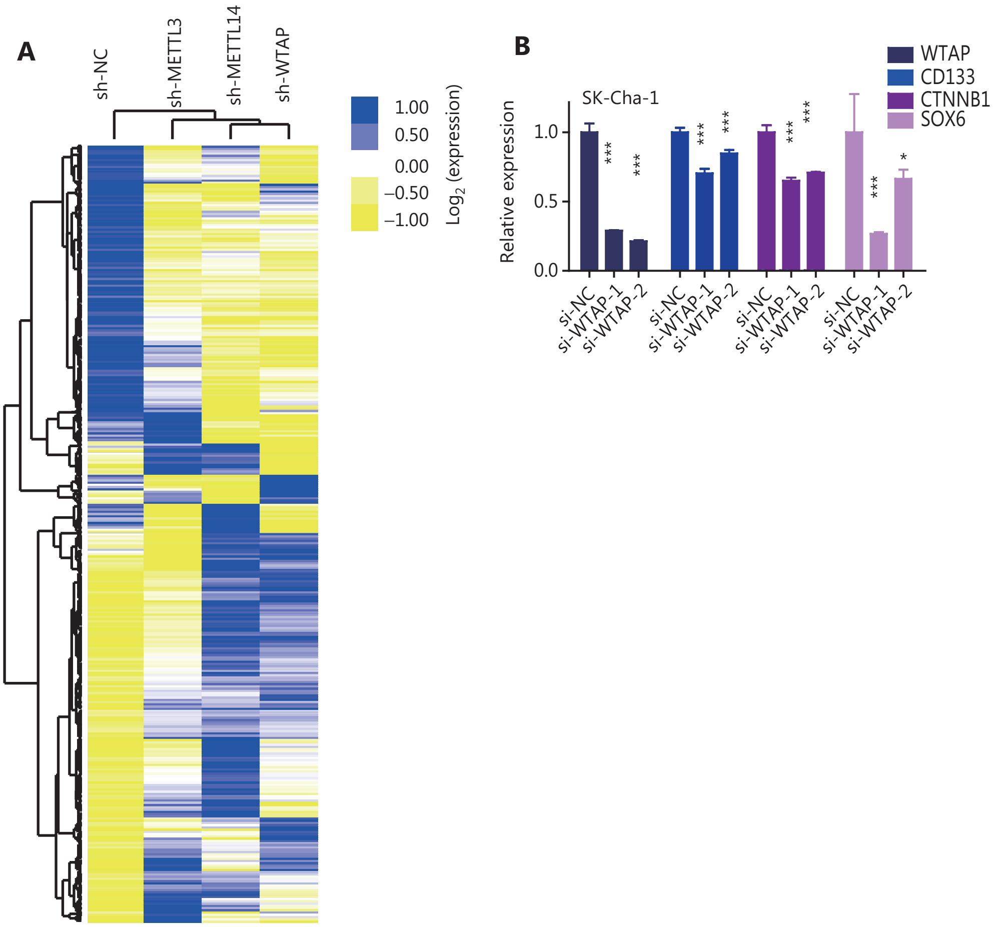

Figure S4 The m6A genes modulated the expressions of stemness-related genes in cholangiocarcinoma (CCA) cells. (A) Cluster analysis of aberrantly expressed genes in MELTT3, METTL14, and WTAP knockdown samples. (B) The qRT-PCR showing the expression levels of METTL3, CD133, CTNNB1, and SOX6 in WTAP-downregulated CCA cells. Error bars denote ± SEM (**P* < 0.05; ****P* < 0.001) in 3 independent experiments.

A

GSE90642 for m6A peak enrichment of METTL14 KO

gene	m6A_FC	m6A_log2Fc
STAT3	0.194791	–2.36
SOX4	1.337	0.419
MYC	0.915734	–0.127
FOXP4	0.516557	–0.953
FOXO3	0.432269	–1.21
FOXO1	0.644387	–0.634
FOXN3	1.314032	0.394
CTNNB1	0.571965	–0.806
FOXE1	0.45376	–1.14

C

GSE90639 for IGF2BP2 RIP-seq

Binding gene	log2_fold-entichement
SOX6	–
SOX4	–
MYC	5.82561
HOXB9	4.9641
HOXA9	3.28792
HOXA6	–
HOXA5	–
HOXA3	4.0679
HOXA10	2.19696
FOXP2	4.72502
FOXP3	–
FOXO1	–
CTNNB1	3.47956
TTF2	3.05486
STAT3	–
FOXP4	–

D

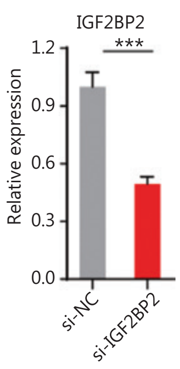

B

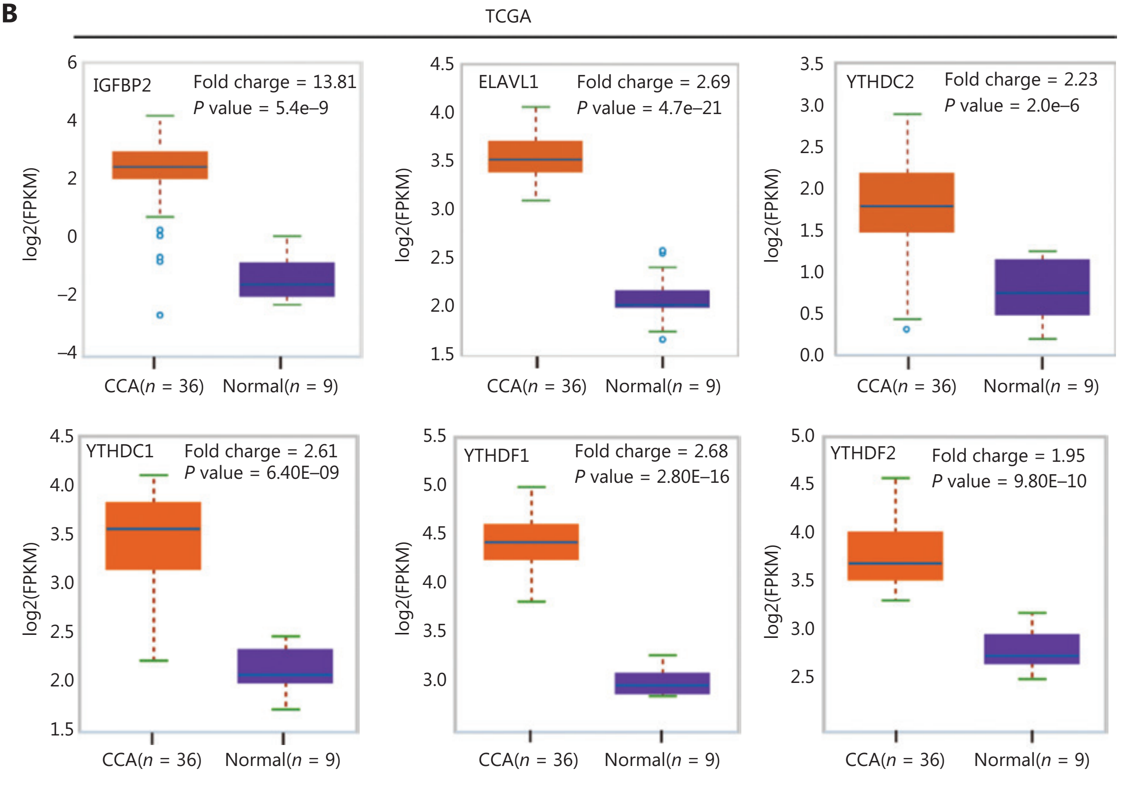

Figure S5 IGF2BP2 improved the RNA stability and translation of stemness-related genes. (A) The GSE90642 data set showing the enrichment of m6A modifications of some stemness-related genes between METTL14 silenced and control cells. (B) The expression patterns of IGF2BP2, ELAVL1, YTHDC2, YTHDC1, YTHDF1, and YTHDF2 in The Cancer Genome Atlas datasets consist of 36 CCA and 9 healthy samples. (C) The GSE90639 data set showing the IGF2BP2-enrichment of many stemness-related genes. (D) The qRT-PCR showing the expression levels of IGF2BP2 in the IGF2BP2 knockdown cells. Error bars denote ± SEM (**P < 0.01; ***P < 0.001) in 3 independent experiments.

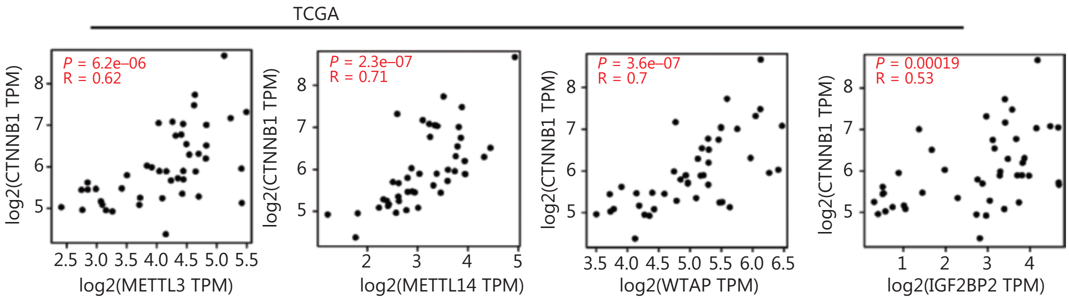

Figure S6 The expression correlations of m6A genes and CTNNB1. Scatter plot showing the correlation of expression between m6A genes and CTNNB1 in the TCGA database of CCA.
